# Assessment of Future Water Yield and Water Purification Services in Data Scarce Region of Northwest China

**DOI:** 10.3390/ijerph18178960

**Published:** 2021-08-25

**Authors:** Xu Yang, Ruishan Chen, Guangxing Ji, Chao Wang, Yuanda Yang, Jianhua Xu

**Affiliations:** 1 Institute of Geographical Sciences, Henan Academy of Sciences, Zhengzhou 450052, China; yx1989422@126.com (X.Y.); wangchao@igs-has.cn (C.W.); 2 Collaborative Innovation Center for Geographic Information Technology of Wisdom Central Plain, Zhengzhou 450052, China; 3 School of Design, Shanghai Jiaotong University, Shanghai 200240, China; chenrsh04@gmail.com; 4 The College of Resources and Environmental Sciences, Henan Agricultural University, Zhengzhou 450002, China; guangxingji@henau.edu.cn; 5 The College of Resources and Environment, Henan University of Economics and Law, Zhengzhou 450046, China; yangyuanda001@163.com; 6 Key Laboratory of Geographic Information Science (Ministry of Education), East China Normal University, Shanghai 200241, China; 7 Research Center for East-West Cooperation in China, East China Normal University, Shanghai 200241, China; 8 School of Geographic Sciences, East China Normal University, Shanghai 200241, China

**Keywords:** water yield service, water purification service, RCP scenarios, data-scarce region, northwest China, arid area

## Abstract

Water shortage and pollution have become prominent in the arid regions of northwest China, seriously affecting human survival and sustainable development. The Bosten Lake basin has been considered as an example of an arid region in northwest China, and the Integrated Valuation of Ecosystem Services and Tradeoffs (InVEST) model has been used to quantitatively evaluate the future water yield and water purification services for four representative concentration pathways (RCP) scenarios. The results show that for the four RCP scenarios, the annual average precipitation in 2020–2050 decreases compared to that in 1985–2015; the area of cultivated land and unused land decreases, and the area of other land-use types increases from 2015 to 2050. The water yield service reduces, while the water purification service increases from 2015 to 2050 in the Bosten Lake basin. In 2050, the water yield and water purification services are the best for the RCP6.0 scenario, and are the worse for the RCP4.5 scenario and RCP8.5 scenario, respectively. The distribution of the water yield and water purification services show a gradual decline from northwest to southeast.

## 1. Introduction

Ecosystem services are the environmental conditions and public utilities that form and sustain human survival, life, and production in ecosystems and their processes [1]. Water ecosystem services are a type of ecosystem services [2,3] and are particularly important in arid and semi-arid areas where water is the key ecological factor. As a typical representative of arid regions in the world [4], the arid regions of northwest China have long been able to maintain the balance among human survival, development, and ecosystems [5]. However, due to global warming and intensified human activities, the fragility of the water ecosystem has increased [6], the service functions of the water ecosystem have been declining, and problems such as the drying up of rivers, disappearance of wetlands, drop in groundwater levels, and water pollution have appeared [7,8,9]. The rapid global socio-economic development (expansion of industrial land, increase in the use of fossil fuels, destruction of natural vegetation, strengthening of agricultural activities, land degradation, etc.) change the absorption of greenhouse gas, ground reflectivity, and evaporation which will lead to regional climate changes. It is estimated that by 2050, rising temperature in northwest China will cause a 27.2% decrease in the area of glaciers and a severe shortage of water for drinking and irrigation [10]. The quantity and quality of water are important resource parameters that need to be managed and are closely related to the ability of the water ecosystem to provide services for human and sustainable development.

The assessment of water ecosystem services is a key issue in the field of hydrology and watershed management [11]. Several methods are currently available to assess water ecosystem services [12]. Although many studies on water ecosystem services were conducted in northwest China, including water yield and water purification services [13,14,15,16,17,18], they have paid less attention to research on the condition of these services in the future and areas with limited data. Due to the complex climate and geographical environment, it is difficult to investigate and monitor these areas, resulting in the collection of limited data. However, data-scarce areas are also an indispensable part of natural ecological environmental research, so it is important to solve the problems of these areas with limited data. With the development of the geographic information system (GIS) and remote sensing (RS), many datasets (satellite imagery data, aerial photography data, data assimilation products, etc.) and methods (e.g., statistical downscaling method) have been applied to solve the problem of lack of data, and models have been proposed to simulate hydrological processes, such as the soil and water assessment tool (SWAT) [19,20], the precipitation runoff modeling system (PRMS) [21], the hydrological simulation program Fortran (HSPF) [22], and the Integrated Valuation of Ecosystem Services and Tradeoffs (InVEST) [23]. The InVEST model has been widely used to assess water ecosystem services [24,25,26,27,28], this model requires less input data and parameters, and can be better applied to the research on water ecosystem services in areas where data is lacking, but most of the previous studies that used the InVEST model were in historical periods; studies on future periods are lacking.

As a typical representative of the arid region in northwest China, the Bosten Lake basin has a complex climate and rapid socio-economic development. The water ecosystem problems, such as water shortage and water pollution, are prominent in this region [29,30,31,32]. This study simulates and analyzes water yield and water purification services for RCP scenarios in the Bosten Lake basin. The major objectives of this study are to determine (1) the spatio-temporal characteristics of water yield and water purification service for the RCP2.6, RCP4.5, RCP6.0, and RCP8.5 scenarios, (2) change in the water yield and water purification services between the historical period and the future period, and (3) effect of precipitation and land-use change on the water yield and the water purification services in the future period. The statistical downscaling method and InVEST model have been used to the research on the future scenario in the Bosten Lake basin in this study. This study contributes to water resources management in the arid regions of northwest China, and provides new ideas for future water ecosystem services in areas with limited data.

## 2. Study Region

**Figure 1:**
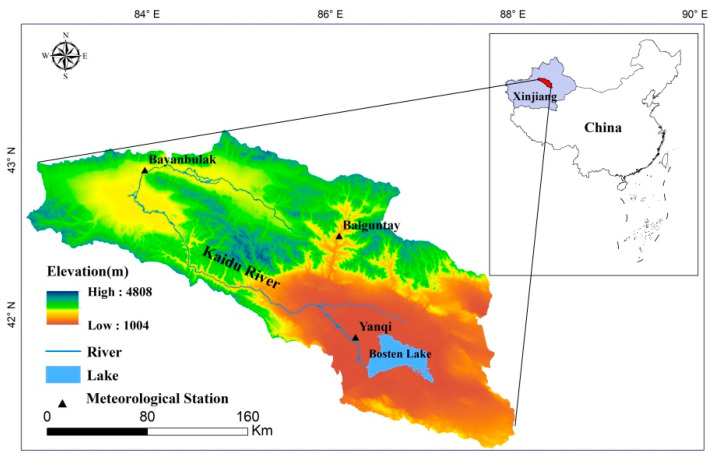
The geographical location of the Bosten Lake basin.

The Bosten Lake basin (82°58′ E–86°05′ E, 42°14′ N–43°21′ N) is located on the southern flank of the Tianshan Mountains in the Xinjiang Uyghur Autonomous Region of China (Figure 1). The surrounding mountains prevent moist air from entering this region, leading to a temperate continental arid type of climate. The Kaidu River originates in the Bayanbulak Grassland, flows through the Yanqi Basin, and finally pours into the Bosten Lake, which is the largest inland freshwater lake in China [33]. The basin is located deep in the hinterland of Eurasia, which has abundant sunshine and strong solar radiation. The average annual temperature is between −6.5 °C and 11.5 °C, the annual precipitation is between 70 mm and 600 mm, and the evaporation is between 500 mm and 1500 mm; there is a difference in the climate between the mountainous and plain areas.

## 3. Materials and Methods

### 3.1. Data Source and Pretreatment

The datasets used in this study include the digital elevation model (DEM), land-use and climate data, soil data, reference evapotranspiration data, root restricting layer depth, the fraction of water available to plants, and watersheds. The DEM data used is the 90 m resolution data of the Shuttle Radar Topography Mission (SRTM), which was downloaded from the United States Geological Survey (USGS) [34]. The 1 km resolution land use data of 2015 was downloaded from the Resource Environmental Science and Data Center of Chinese Academy of Sciences [35], and the data of 2050 was downloaded from Department of Earth System Science of Tsinghua University [36,37]. The 0.625° × 0.5° resolution reanalysis precipitation and temperature data of the Modern-Era Retrospective analysis for Research and Applications, version 2 (MERRA-2), were downloaded from the National Aeronautics and Space Administration (NASA) [38]. The 0.125° × 0.125° resolution sunshine duration data of ERA-Interim datasets were downloaded from the European Centre for Medium-Range Weather Forecasting (ECMWF) [39]. The future sunshine duration data have been calculated based on the average years of the historical sunshine duration. The reference evapotranspiration has been calculated using the Hamon equation [40]. The 1 km resolution root-restricting layer depth was obtained from the Harmonized World Soil Database (HWSD) provided by the Food and Agriculture Organization (FAO) [41,42]. The 1 km resolution for a fraction of water available to plants has been calculated using the empirical formula from the Plant Available Water Content (PAWC) and the HWSD data [43]. The data on watersheds is vector data and was obtained from the DEM data using the ArcSWAT software. The input data of the InVEST model includes many parameters related to vegetation and soil, which were obtained from the relevant studies and the InVEST User Guide. Individual parameters were adjusted through sensitivity analysis. The future precipitation and temperature data with 0.5° × 0.5° resolution include five main global climate model (GCM) results of the Coupled Model Intercomparison Project-Phase 5 (CMIP5) [44], and these data were downloaded from The Inter-Sectoral Impact Model Intercomparison Project (ISI-MIP) [45,46,47,48], including four representative concentration paths (RCP) scenarios (RCP2.6, RCP4.5, RCP6.0, and RCP8.5), which represent four greenhouse gas concentration scenarios for assessing the future climate. Different RCP scenarios are arranged from low to high as RCP2.6, RCP4.5, RCP6.0, and RCP8.5, where the latter number indicates the radiative forcing level from 2.6 W/m^−2^ to 8.5 W/m^−2^ by 2100. The RCP2.6 scenario represents the scenario in which human beings vigorously implement energy-saving and emission reduction measures to control the increase in greenhouse gas concentration in the future; RCP8.5 scenario represents the increase in greenhouse gas concentration where no emission reduction measures are taken at all; RCP 4.5 and RCP 6.0 scenarios are between the two scenarios.

Since there were very few meteorological stations, the interpolation method was not suitable for the study region. The precipitation and temperature data of data assimilation products could be used for the study region, but the resolution required improvement. After analysis and comparison [49,50,51], we selected the statistical downscaling method to address the problem of sparse data. The spatial and temporal characteristics of the regional climate can be revealed by improving the resolution of the reanalysis data and combining it with topographic data [52]. According to the existing downscaling methods and regional characteristics, an improved downscaling method that can dynamically adjust parameters according to terrain characteristics was selected, which was verified in the study area and could effectively solve the spatial resolution and accuracy of the data [53]. The downscaling method comprises three steps: simulation of precipitation gradients and temperature lapse rates, downscaling of the reanalysis climate data, and calculation of high-resolution precipitation and temperature data.

The downscaling equations (Equations (1) and (2)) are as follows:

(1) $$\;p_{d}=p_{r}+\left(h_{s}-h_{r}\right)\cdot \left[a\left(h_{s}+h_{r}\right)+b\right]$$

(2) $$t_{d}=t_{r}+\left(h_{s}-h_{r}\right)\cdot m_{\;}$$

where *p_d_* and *t_d_* are the statistical downscaling precipitation and temperature, respectively, and *p_r_* and *t_r_* are the reanalysis precipitation and temperature data, respectively. The parameters *m*, *a*, and *b* are coefficients for the equations and have been estimated from meteorological observations. The altitude of STRM is denoted as *h_s_*, and the elevation of the reanalysis data *h_r_* has been calculated using a bilinear interpolation resampling method.

There are many methods to calculate reference evapotranspiration, including the Penman–Monteith equation [54], the improved Hargreaves equation [55], the Hamon equation [40,56], and the method for evaluating reference evapotranspiration when the pan evaporation is known [42]. In this paper, the Hamon equation has been used to calculate the reference evapotranspiration of the Bosten Lake basin. The equation used for the calculation is as follows (Equation (3)):

(3) $$PED_{Hamon}=13.97dD^{2}W_{t}$$

where *PED_Hamon_* is the reference evapotranspiration, *d* is the number of days in a month, *D* is the average number of hours of sunshine per year (in 12 h units), and *W_t_* is the saturated vapor density.

The fraction of water available to plants has been calculated by the empirical formula of the PAWC (Equation (4)), which is as follows:

(4) $$PAWC\left(x\right)=54.509-0.132sand-0.003{\left(sand\right)}^{2}-0.005silt-0.006{\left(silt\right)}^{2}-0.738clay+0.007{\left(clay\right)}^{2}-2.688OM+0.501{\left(OM\right)}^{2}$$

where *sand* is the percentage of sand in the soil, *silt* is the percentage of silt in the soil, *clay* is the percentage of clay in the soil, and *OM* is the percentage of organic matter in the soil.

### 3.2. Research Methods

In the InVEST model, the water yield (WY) and nutrient delivery ratio (NDR) components are designed to map the spatial distribution of water in a basin and the source and transport process of nutrients across a basin. The WY and NDR estimate the quantity of water yield and the quantity of nitrogen and phosphorus at each watershed in the area of interest, respectively. The InVEST model calculates the total water yield, nitrogen export, and phosphorus export in the ecosystem. In the WY component, input data require watersheds, precipitation, land use, DEM, depth to root-restricting layer, reference evapotranspiration, and fraction of water available to plants; parameters require plant evapotranspiration coefficient for each land use or land cover class. In the NDR component, input data require watersheds, precipitation, land use, DEM; parameters require threshold flow accumulation, and nutrient load, retention efficiency, critical length for each land use or land cover class.

The WY component is based on the Budyko curve, where the annual water yield is determined as follows (Equation (5)):

(5) $$Y\left(x\right)=\left(1-\frac{AET\left(x\right)}{P\left(x\right)}\right)\cdot P\left(x\right)\;$$

where *x* is the each pixel on the landscape, *Y(x)* is the annual water yield (m^3^), *AET(x)* is the annual actual evapotranspiration for pixel *x*, and *P(x)* is the annual precipitation for pixel *x*.

The NDR component uses the mass conservation method to simulate the transfer of nutrients in space. The equations to determine nutrients (Equations (6) and (7)) are as follows:

(6) $$X_{expton}=\underset{i}{\sum }X_{expi}$$

(7) $$X_{expi}=load_{surf,i}\cdot NDR_{surf,i}+load_{subs,i}\cdot NDR_{subs,i}$$

where *X_expton_* is the total export amount of nutrients in the river basin (kg/yr) and *X_expi_* is the export amount of each grid of nutrients (kg/yr). The *load_surf,i_* is the surface nutrient load (kg/ha·yr^−1^), *NDR_suf,i_* is the surface nutrient transfer rate, *load_subs,i_* is the subsurface nutrient load (kg/ha·yr^−1^), and *NDR_subs,i_* is the subsurface nutrient transfer rate.

Spatial analysis has been performed in this study using GIS. The raster calculation tool, reclassification tool, zonal statistics tool, etc., in the ArcGIS software, have been used to analyze the spatio-temporal dynamic processes of water ecosystem services, climate and land-use change, and the impact of climate and land-use change on water ecosystem services.

## 4. Results

### 4.1. Distribution of Land Use in 2050 for the RCP Scenarios

**Figure 2:**
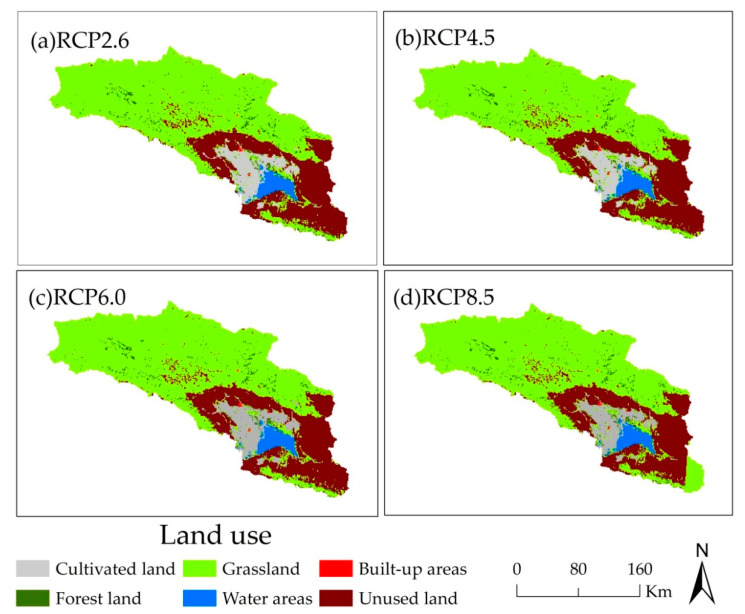
The distribution of land use of Bosten Lake basin in 2050 for RCP scenarios.

Land use in the Bosten Lake basin includes cultivated land (CL), forest land (FL), grassland (GL), water areas (WA), built-up areas (BA), and unused land (UL). In 2050, land use for the RCP scenarios shows spatial heterogeneity, and the spatial distribution of land use among the four RCP scenarios is similar (Figure 2). Cultivated land and built-up areas are distributed mainly in the vicinity of the lake. There is limited forest land, and this is scattered across the basin. Grassland, and the various land types classified as unused land, is widely distributed in the basin, with grassland predominating in the west, and unused land mainly in the east, especially the unused land, is dominated by the Gobi and bare rocky gravel. Water areas are distributed in the southeast of the basin, with lakes in the plains.

**Figure 3:**
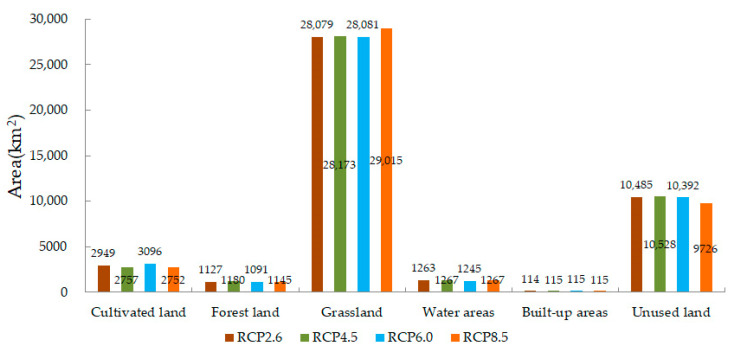
Land use area of Bosten Lake basin in 2050 for RCP scenarios.

Figure 3 shows that for the four RCP scenarios in the Bosten Lake basin in 2050, cultivated land, forest land, grassland, water areas, built-up areas, and unused land account for approximately 67%, 2.5–2.7%, 64–66%, 3%, 0.26%, and 23% of the whole basin area, respectively. As the level of radiative forcing increases, i.e., the concentration of greenhouse gas increases, the area of cultivated land and unused land in the Bosten Lake basin decrease, while the forest land, grassland, water areas, and built-up areas increase (Figure 3). Compared to the land use in 2015 in the Bosten Lake basin, the cultivated land, forest land, and grassland increase, while water areas, built-up areas, and unused land decrease [57]. This is related to the increased melting of snow due to an increase in the temperature and the concentration of greenhouse gas, the growth of natural vegetation caused by the snow melting, and the deterioration of the environment leading to unsuitability for the habitation of humans.

### 4.2. Precipitation Changes from 2020 to 2050

**Figure 4:**
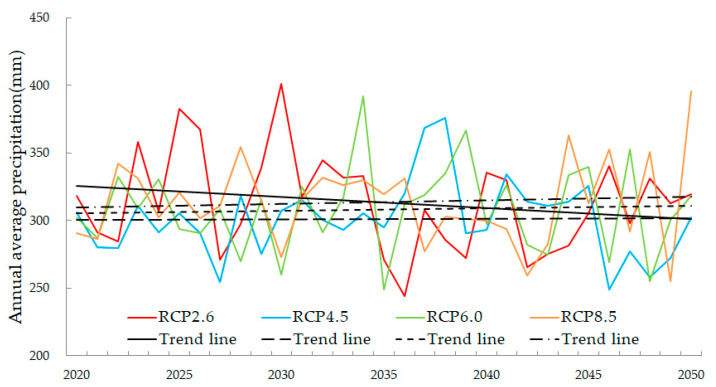
The annual average precipitation of the Bosten Lake basin for RCP scenarios from 2020 to 2050.

The statistical downscaling method used in this study was based on the relationship between precipitation and altitude, and the topography in the region generally shows no change. Therefore, this method can still downscale future climate data to obtain precipitation with a resolution of 1 km × 1 km data. According to the simulations, the annual average precipitation of the Bosten Lake basin fluctuates between 243.94 mm and 401.12 mm from 2020 to 2050 (Figure 4). The overall precipitation decreases, compared to the historical periods [57,58], which is related to the increase in temperature caused by the increase in greenhouse gas concentration. The increase in temperature intensifies evaporation leading to higher evaporation of water. The precipitation relatively declines from the perspective of water balance. The annual average precipitation for the four RCP scenarios shows high fluctuations in the next 30 years, but the changes are different. The rate of change of annual average precipitation for the RCP2.6, RCP4.5, RCP6.0, and RCP8.5 scenarios are −0.0361 mm/yr, 0.0017 mm/yr, 0.0075 mm/yr, and 0.012 mm/yr, respectively.

**Figure 5:**
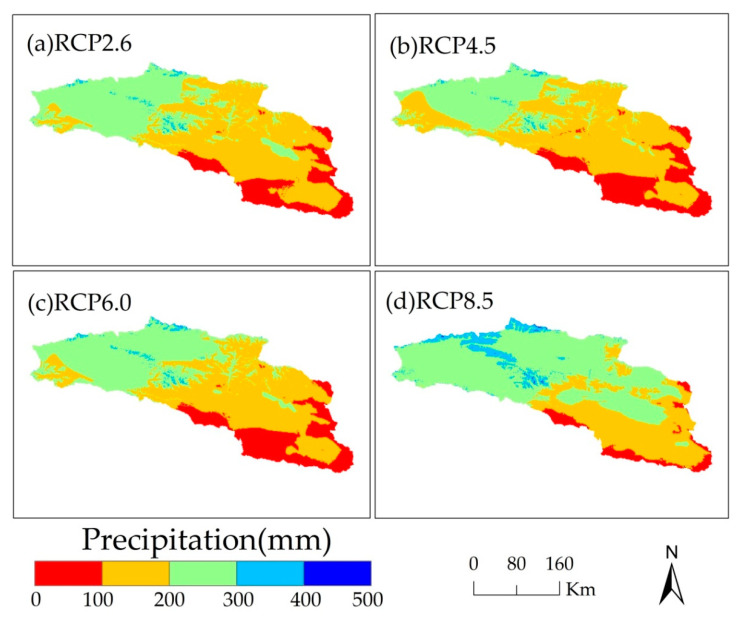
The spatial distribution of annual average precipitation of Bosten Lake basin for RCP scenarios in 2050.

For the four RCP scenarios, the annual average precipitation in the Bosten Lake basin gradually decreases from the northwest to the southeast in 2050 (Figure 5). With the increase in the concentration of greenhouse gas, areas with high precipitation gradually increase, and areas with low precipitation gradually decrease. The area with high precipitation is small, and this is consistent with the feature of low rainfall in northwest China.

### 4.3. Spatio-Temporal Distribution of Water Yield, Nitrogen Export, and Phosphorus Export

The parameter schemes of the WY and NDR modules in the InVEST model have been determined by the biophysical properties of the vegetation and soil and generally show no change. Therefore, the parameter schemes of the two modules in the historical period are also suitable for the simulation of future scenarios.

In 2050, for the RCP2.6 to RCP8.5 scenarios of the Bosten Lake basin, the water yield, nitrogen export, and phosphorus export decrease by 0.2 × 10^8^ m^3^, 262.05 t, and 108.33 t, respectively (Table 1). The nitrogen export and phosphorus export are still large, while the proportion of nitrogen and phosphorus export decrease. The water yield in 2050 for the four RCP scenarios is small.

**Figure 6:**
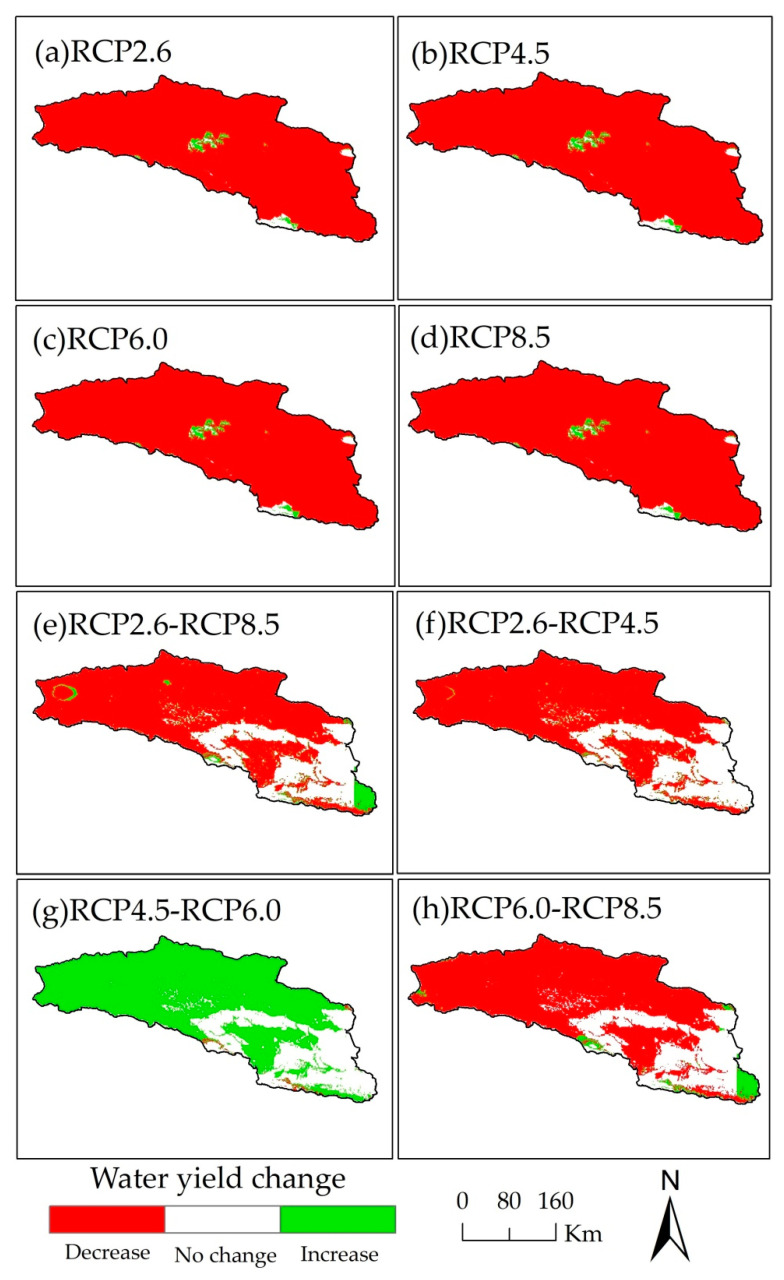
The distribution of water yield change of Bosten Lake basin in 2050 for RCP scenarios and in 2015 (**a**–**d**). The distribution of water yield change of Bosten Lake basin between different RCP scenarios in 2050 (**e**–**h**).

In the Bosten Lake basin, most areas that yield water decrease, and parts that yield water in mountainous areas increase between 2015 and 2050 (Figure 6a–d). With the increase in greenhouse gas concentration, water yield change shows that the overall decrease in water yield and the areas yielding water fluctuate. The water yield for the RCP4.5 scenario is the least, while that for the RCP6.0 scenario is the most (Figure 6e–h).

**Figure 7:**
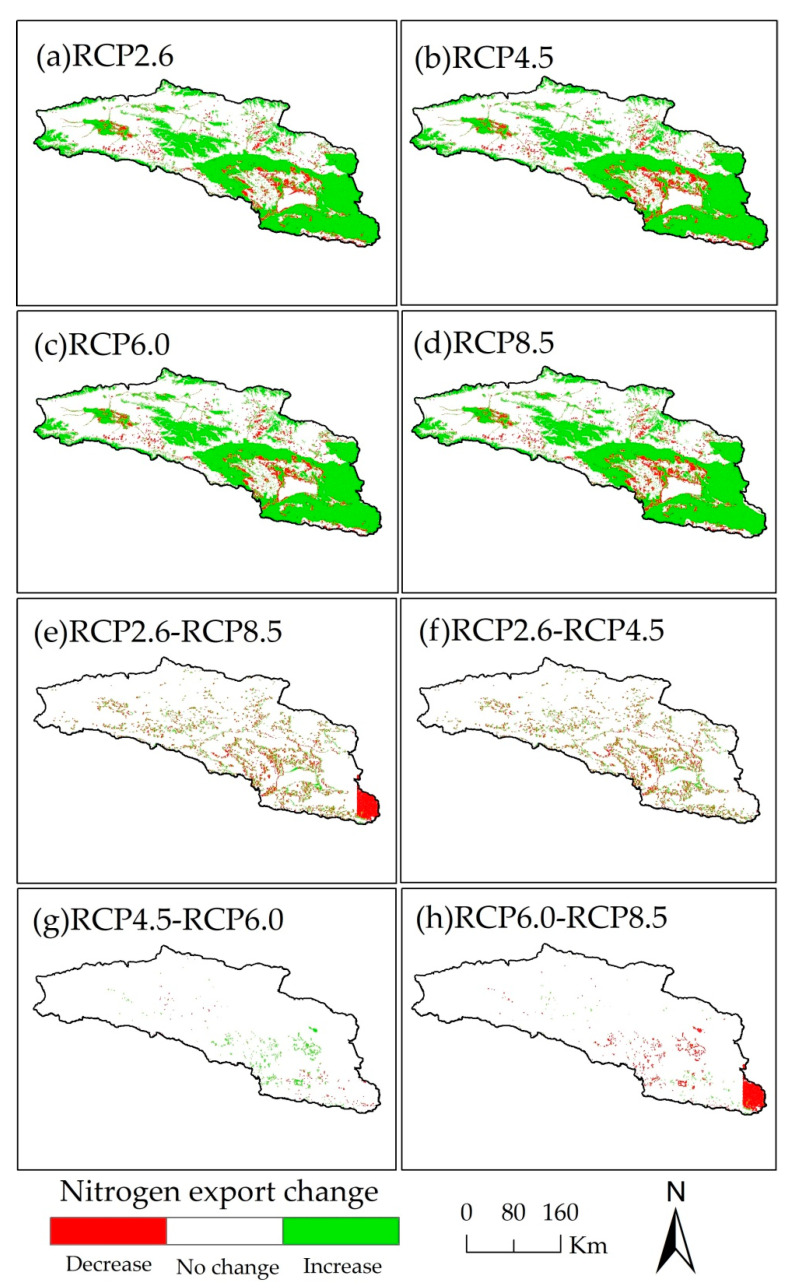
The spatial distribution of nitrogen export change of Bosten Lake basin in 2050 for RCP scenarios and in 2015 (**a**–**d**), between different RCP scenarios in 2050 (**e**–**h**).

**Figure 8:**
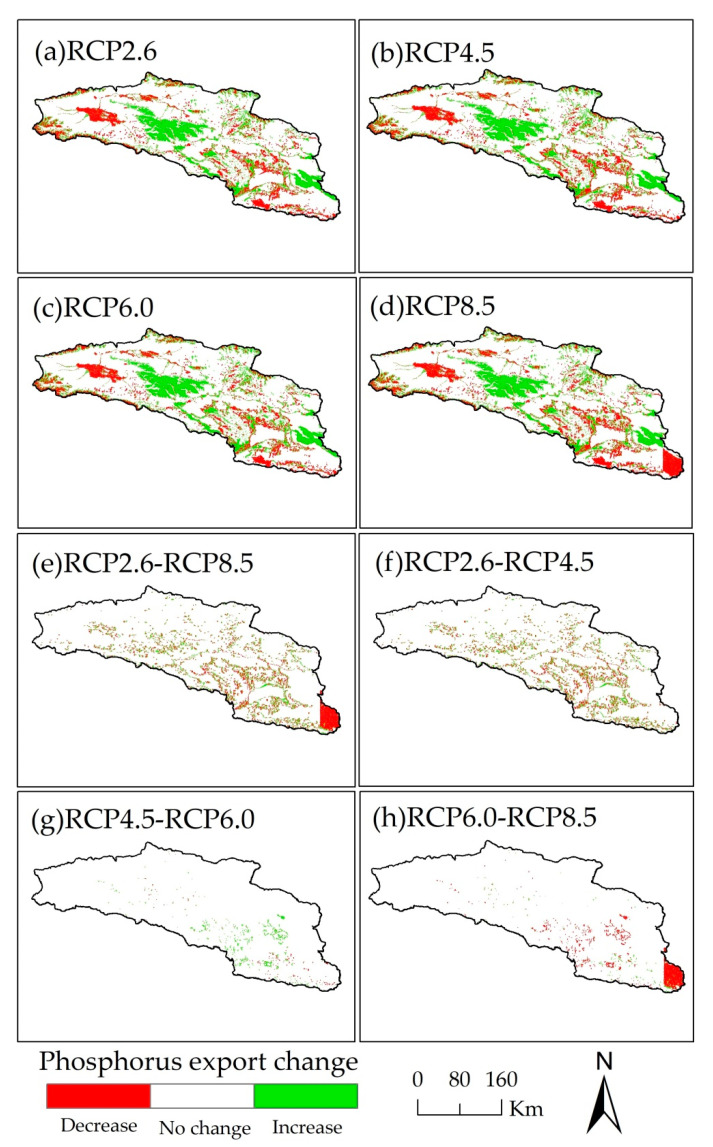
The spatial distribution of phosphorus export change of Bosten Lake basin in 2050 for RCP scenarios and in 2015 (**a**–**d**), between different RCP scenarios in 2050 (**e**–**h**).

As the concentration of greenhouse gas increases, the nitrogen and phosphorus export varies widely in the whole basin. The spatial changes in nitrogen and phosphorus export between 2015 and 2050 for the RCP scenarios are large in the Bosten Lake basin, and the nitrogen export increases in a large area, while the change in phosphorus export is scattered in the basin. In the Bosten Lake basin, the areas where nitrogen and phosphorus export change are distributed throughout the basin (Figure 7a–d and Figure 8a–d). Among the four RCP scenarios, there is no large-scale change in the nitrogen and phosphorus export, and the change occurs mainly in the southeastern plains, as well as the mountainous areas in the middle of the Bosten Lake basin (Figure 7e–h and Figure 8e–h).

### 4.4. The Impact of Land Use and Precipitation Change on Water Yield, Nitrogen Export, and Phosphorus Export

As the snow melts in the future due to higher temperatures, precipitation becomes the main source of water in the Bosten Lake basin. Due to the socio-economic pattern in the Bosten Lake basin, the sources of nitrogen and phosphorus are mainly agricultural and domestic pollutants. The vegetation and soil influence the water yield and water purification.

**Figure 9:**
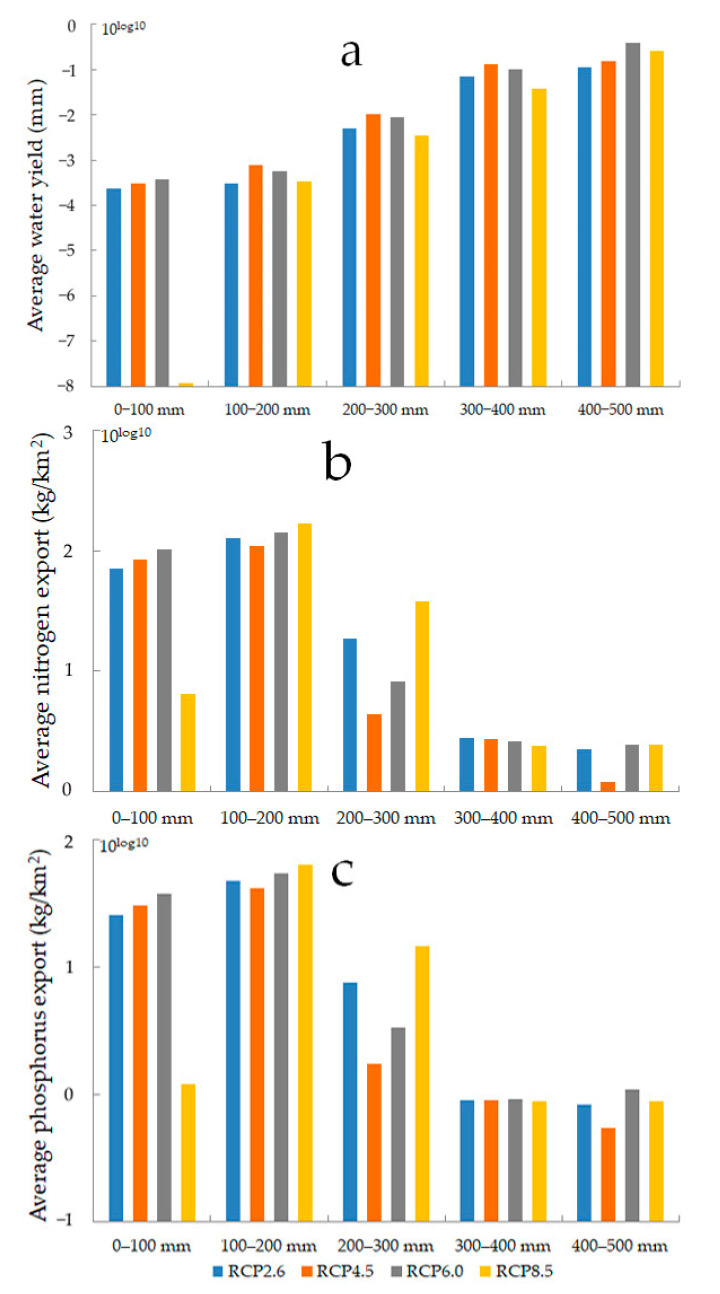
Average water yield (**a**), average nitrogen export (**b**) and average phosphorus export (**c**) of different precipitation in 2050 for RCP scenarios.

Precipitation has been classified into five categories, *viz*. 0–100 mm, 100–200 mm, 200–300 mm, 300–400 mm, and 400–500 mm. With the increase in precipitation, the average water yield increases, and the average nitrogen and phosphorus export fluctuate and decrease overall for the RCP scenarios, reaching the maximum value for precipitation between 100 mm and 200 mm (Figure 9). Regarding the spatial distribution of precipitation in the future, the two water ecosystem services in the mountainous areas remain better than those in the plains. Therefore, the water ecological environment not only maintains the current situation in the mountainous areas but also strengthens the protection and restoration of the plains. The response of the average water yield and those of the average nitrogen and phosphorus export show opposite effects to precipitation change, which indicates that there is a tradeoff between the water yield service and the water purification service in response to precipitation change.

**Figure 10:**
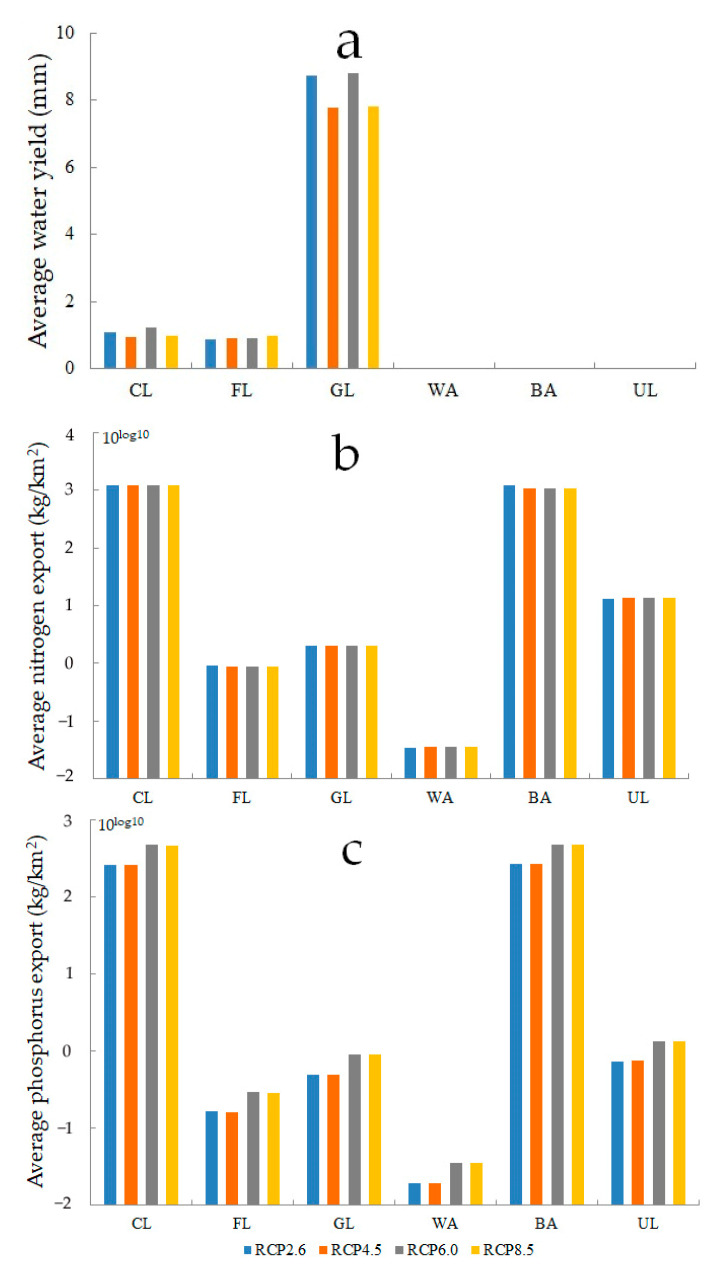
Average water yield (**a**), average nitrogen export (**b**) and average phosphorus export (**c**) of different land use types in 2050 for RCP scenarios.

The rapid socio-economic development causes an increase in greenhouse gas concentration, leading to future land-use change through climate change, which affects water resources and nutrients by land-use change. For the four RCP scenarios in 2050, water yield only occurs in cultivated land, forest land, and grassland in the Bosten Lake basin; the largest and smallest values are for the grassland and forest land, respectively (Figure 10a). The RCP6.0 and RCP8.5 scenarios have high values of average water yield in the three land-use types, while the RCP2.6 and RCP4.5 scenarios have low values of average water yield (Figure 10a). The minimum value of average nitrogen and phosphorus export are in the water areas, while the maximum value of the average nitrogen export for the four RCP scenarios and the average phosphorus export for the RCP2.6 scenario are in cultivated land, but the maximum value of the average phosphorus export for the other three RCP scenarios are in built-up areas (Figure 10b,c). The average nitrogen and phosphorus export in the six land-use types are inconsistent for the different RCP scenarios (Figure 10b,c).

## 5. Discussion

### 5.1. Future Trend in Water Yield, Nitrogen Export, and Phosphorus Export

The parameters are related to biophysical properties of the underlying surface structure, which is difficult to change in the same area. The parameters of future simulations are based on the parameters of historical schemes, which have been verified in the previous studies on water yield, nitrogen export, and phosphorus export in the Bosten Lake basin [57,58]. The decrease in water yield and the increase in nitrogen and phosphorus export between 2015 and 2050 indicate that the future water yield and water purification service functions decline. This further exacerbates the contradiction between water supply and demand and aggravates water pollution in the water-scarce Bosten Lake basin. We found that the future water yield service is better for the RCP6.0 scenario and worse for the RCP4.5 scenario; the water purification service is better for the RCP8.5 scenario and worse for the RCP6.0 scenario (Table 1). The water yield service is mainly affected by precipitation, but the water cycle process is affected by the underlying surface structures, resulting in the inhibition or promotion of water yield by land use, and the changes in water yield for the RCP scenarios are inconsistent with the changes in precipitation. Water purification service is mainly affected by cultivated land, which is consistent with cultivated land changes for the RCP scenarios. This is in line with previous studies on the response of water yield service and water purification service to climate and land-use change [59]. The restoration of soil and vegetation increases infiltration and evapotranspiration, improves the ability to purify pollutants, and decreases impervious surfaces, resulting in a decrease in water yield, nitrogen export, and phosphorus export. Otherwise, an increase in water yield, nitrogen export, and phosphorus export occur.

Human activities such as agricultural development and the use of fossil fuels have increased the concentration of greenhouse gas, which has caused the warming in the Bosten Lake basin, and the increased evaporation has led to the melting of snow in the mountainous areas of the basin. This is consistent with the Chinese second glacier catalog statistics, indicating that increasing global temperature, caused by greenhouse gas emissions, is the main reason for the melting of snow and glaciers. Precipitation is the main source of water in the Bosten Lake basin. In the future, the water yield depends more on precipitation which causes a more serious water shortage in the study region that receives little precipitation. A large amount of water from the melting of snow promotes the conversion of unused land into grassland in mountainous areas. With human efforts to implement energy-saving and emission-reduction measures, the forest land and grassland increase for the RCP scenarios. The increase in vegetation reduces water yield and nitrogen and phosphorus export. However, the increase in vegetation is mainly in the mountains, with little nitrogen and phosphorus export, while the nitrogen and phosphorus export in the Bosten Lake basin is in the southeastern plains. Thus, the impact of the increase in vegetation on nitrogen and phosphorus export is relatively small. The agricultural development in the basin promotes the expansion of cultivated land, and agricultural irrigation causes water loss. This increases the use of fertilizers and pesticides, which reduces water yield and increases nitrogen and phosphorus export. The agriculture is the main source of nitrogen and phosphorus export, because it is the main source of economic development in the Bosten Lake basin. The built-up areas decrease because of agricultural development, increase in vegetation, the unsuitability for living due to greenhouse gas emissions, and future human energy-saving and emission-reduction measures, which reduce water yield, and nitrogen and phosphorus export. However, built-up areas, which account for a small proportion of areas, have relatively little impact on the water yield and nitrogen and phosphorus export in the Bosten Lake basin.

### 5.2. Attribution of Water Ecosystem Services and Implications for Water Resource Policies

With the rapid socio-economic development, an increase in greenhouse gas emissions causes worse water yield and water purification services in the future Bosten Lake basin. The future situation needs to be considered seriously, and humans should take appropriate measures to prevent the deterioration of the water ecological environment in the arid regions of northwest China. Although the water ecological environment policies have historically been implemented in the arid regions of northwest China, it is difficult for the government to formulate policies for development in the future. Through the quantitative assessment of future water ecosystem services, several recommendations have been provided for the future water ecological protection of the basin.

Responding to climate change in advance and improving the management of water resources should be considered. First, the shortage of water resources in arid regions, especially the effective interception of precipitation in plain areas, can solve the problem of water use. Second, storage of water sources, such as building dams and reservoirs, excavating canals to control and deploy water resources, and drawing water from relatively abundant areas to scarce areas can be implemented. Third, projects for biological or physical protection along the lower reaches of rivers and lakes, as well as point source and non-point source pollution control projects, should be undertaken to protect the water sources.

The natural vegetation needs to be improved, and an increase in the mix of forest land and grassland is required. Under the premise of protecting the original natural forests and grasslands, more conservation forests and water and soil conservation forests, which play an important role in the water supply and water purification, have to be added to the plain areas of arid regions especially oasis areas. The scientific and well-planned construction of a mix of forest land and grassland increases water supply and reduces the output of pollutants.

Protecting the quality of water resources, improving the control of pollutants, and sewage treatment can be performed. First, agriculture is the main source of economic development in arid regions, and pesticides and fertilizers are the main pollution sources. The rate of use of pesticides and fertilizers should be controlled. By changing the underlying surface, the amount of sewage can be reduced, and the distance that the sewage moves can be increased, thus reducing the amount of irrigation sewage in the water source. Second, investment in sewage treatment can be increased, and the effective use of sewage can be improved. The reprocessing and utilization of sewage can reduce water pressure in arid regions.

The efficiency of the use of water resources can be improved, and measures to manage water resources can be formulated. The economic development and climatic conditions in the arid regions determine the measures that need to be taken to effectively reduce water loss and improve the efficiency of water use. For example, surface canals can be built, and anti-seepage measures can be improved, underground canals can be used, and irrigation can be performed at a certain time to reduce evaporation. If the land is not suitable for farming, it should be converted from farmland to forests and grassland immediately. For example, after preferential treatment of lightly polluted water sources, they are reused as non-drinking water sources for urban green land watering and road watering, etc., and the treatment of production wastewater and domestic sewage meets irrigation standards to relieve the water diversion pressure of rivers and lakes.

### 5.3. Limitations and Prospects

Although this study provides insights into the assessment of water yield and water purification services in the data-scarce arid region of northwest China, there are limitations in the current study. The future predictions of this study are only based on RCP scenarios that focused on greenhouse gas concentration caused by climate change. In the future, predictions for multiple scenarios (e.g., shared socio-economic paths (SSPs) scenario of CMIP6) should be considered. Future data acquisition is based on historical data and forecasting methods, but diversified historical data sources and forecasting methods have an impact on future forecasts. For example, land use changes are affected by a variety of factors, but land use data used in this study are only based on RCP scenarios with climate change, making future predictions uncertain. Only macro simulation and analyses have been performed in this study; microscopic mechanisms have mostly been untouched. Some ecological and water-related physicochemical processes are relatively simple. Other models and methods, combined with the InVEST model, can be used to strengthen the ecological process. Due to a lack of data, some input parameters have been obtained from the official user’s guide and studies using the InVEST model with the sensitivity analysis method. If accurate information of the study region is used to calibrate the input parameters, it can improve the results of the simulations. Field sampling, consulting experts, and cooperation with micro-researchers can solve the problem of the lack of data. The influence of human factors on water ecosystem services should be considered. Socio-economic data is statistically based on administrative units and cannot be used directly in non-administrative areas; the spatialization of the data on socio-economic statistics is an effective solution.

## 6. Conclusions

Multi-source data and methods have been used to evaluate the future water ecosystem services of the inland river basins in the arid regions of northwest China. The results show that as the level of radiative forcing increases, the rate of precipitation also increases; however, land-use change, affected by the climate, has unique characteristics, which leads to differences in water yield and water purification services for different RCP scenarios. In the Bosten Lake basin, there is a decline in the average annual precipitation in 2020–2050 compared to that in 1985–2015, and there is an increase in cultivated land, forest land, and grassland, and a decrease in water areas, built-up areas, and unused land from 2015 to 2050 for four RCP scenarios. The water yield service reduces, while the water purification service increases from 2015 to 2050 in the Bosten Lake basin, but they are both still poor. For the four RCP scenarios, the water yield are arranged from low to high as RCP4.5, RCP8.5, RCP2.6, and RCP6.0, the nitrogen and phosphorus export arranged from low to high as RCP8.5, RCP4.5, RCP2.6, and RCP6.0. It is necessary to maintain the water ecological environment in the mountainous areas of the northwest basin, and strengthen the water ecological restoration and protection of the plain areas in the southeast of the basin. Humans need to change land use to improve water yield and water purification services in the arid regions of northwest China in the face of climate change.

## Figures and Tables

**Table 1:** Water yield, nitrogen and phosphorus export of Bosten Lake basin in 2050 for RCP scenarios.

RCP Scenario	Water Yield (m^3^)	Nitrogen Export (t)	Phosphorus Export (t)
RCP2.6	2.49 × 10^8^	3766.92	1414.19
RCP4.5	2.22 × 10^8^	3518.51	1307.81
RCP6.0	2.51 × 10^8^	3974.48	1499.95
RCP8.5	2.29 × 10^8^	3504.87	1305.86

## Data Availability

Data are contained within the article.
